# Diagnostic accuracy of ultrasonographic features for lymph node metastasis in papillary thyroid microcarcinoma: a single-center retrospective study

**DOI:** 10.1186/s12957-017-1099-2

**Published:** 2017-01-26

**Authors:** Zeming Liu, Wen Zeng, Chunping Liu, Shuntao Wang, Yiquan Xiong, Yawen Guo, Xiaoyu Li, Shiran Sun, Tianwen Chen, Yusufu Maimaiti, Pan Yu, Tao Huang

**Affiliations:** 1 Department of Breast and Thyroid Surgery, Union Hospital, Tongji Medical College, Huazhong University of Science and Technology, Number 1277, Jiefang Road, Wuhan, Hubei Province People’s Republic of China; 2 Department of Ophthalmology, Zhongnan Hospital, Wuhan University, Wuhan, Hubei China; 3 Department of Breast and Thyroid Surgery, Affiliated Nanshan Hospital, Guangdong Medical University, Shenzhen, China

**Keywords:** Ultrasonographic features, Lymph node metastasis, Papillary thyroid microcarcinoma

## Abstract

**Background:**

Whether sonography is an appropriate imaging modality for cervical lymph nodes in patients with papillary thyroid microcarcinoma (PTMC) remains unclear. Hence, this study aimed to evaluate the diagnostic value of ultrasonography (US) features for lymph node metastasis in PTMC.

**Methods:**

Seven hundred twelve patients with PTMC who underwent conventional ultrasonography examinations of the cervical lymph nodes were included. All included cases underwent total thyroidectomy plus prophylactic central lymph node dissection. The included lymph nodes were marked superficially, and the corresponding lymph nodes were completely removed and sent for pathological examination. The US features of lymph nodes with and without metastasis were compared, and the odds ratios of the suspicious US features were determined with univariate and multivariate analyses.

**Results:**

Round shape, loss of an echogenic fatty hilum, cystic change, calcification, and abnormal vascularity were significantly more common in metastatic than nonmetastatic lymph nodes, whereas the boundary and echo did not significantly differ. Multivariate logistic regression analysis showed that round shape, loss of echogenic fatty hilum, cystic change, calcification, and abnormal vascularity were independent predictive factors for the assessment of metastatic lymph nodes. Round shape had the highest sensitivity of all variables, while loss of an echogenic fatty hilum had the highest specificity and accuracy. The area under the receiver operating characteristic curve, which was calculated to verify the relationship between the various US features and metastatic lymph nodes, was 0.793.

**Conclusions:**

Our study found that the US features of round shape, cystic change, calcification, loss of echogenic fatty hilum, and abnormal vascularity were useful sonographic criteria for differentiating between cervical lymph nodes with and without metastasis.

## Background

According to the World Health Organization classification system, papillary thyroid microcarcinoma (PTMC) is defined as a thyroid cancer measuring ≤1.0 cm in its greatest dimension [1]. Reportedly, PTMCs account for approximately 30% of all papillary thyroid cancers (PTCs) [2, 3].

Thyroid cancer, including PTMC, often metastasizes to the cervical lymph nodes, with lymph node metastasis occurring in 37.3% of all cases in one previous study [2]. Lymph node metastasis is the most important risk factor for recurrence and poor overall survival [4, 5]. Therefore, early detection of cervical lymph node metastasis plays an important role for planning the surgery and management of patients with PTMC [6].

Currently, sonography is the modality of choice for providing guidance for fine-needle aspiration biopsy and for imaging of cervical lymph nodes in patients with PTC, for both preoperative and postoperative surveillance [7]. However, whether sonography is also a good choice for cervical lymph node evaluation in patients with PTMC remains unclear. Accordingly, in the present study, we aimed to illustrate the diagnostic value of ultrasonographic (US) features for lymph node metastasis in PTMC.

## Methods

### Patients

Our study included 712 patients with PTMC who underwent conventional US examinations of the thyroid gland and cervical lymph nodes before surgery in Union Hospital, China, from January 2012 to July 2015. Our study was approved by the Ethics Committee of our hospital (name of the ethics committee: Ethics Committee of Union Hospital), and written informed consent was obtained from each patient before the US examination.

All patients evaluated in the study underwent total thyroidectomy plus prophylactic central lymph node dissection. Cases with inconsistent pre- and postoperative diagnoses were excluded, as were PTC patients with tumor size >1 cm. If a patient had multiple suspicious nodes, representative node was evaluated.

### Ultrasonography evaluation

All conventional US examinations were performed by two independent ultrasound physicians with an Acuson S2000 diagnostic US system (Siemens Medical Solutions). The patients were examined in the supine position with a fully exposed neck. The included lymph nodes were marked superficially, and the corresponding lymph nodes were completely removed and sent for pathological examination. Calcifications of lymph nodes have been reported to appear as punctate microcalcifications and to present as psammoma bodies on cytology.

### Statistical analysis

Initial clinical and pathological data were collected by using EpiData Software v3.1 (EpiData Association, Odense, Denmark). All statistical analyses were performed by SPSS software, version 13.0 (SPSS, Chicago, IL), and a two-tailed *P* value of less than 0.05 was considered as statistically significant. Comparisons of frequency distributions were performed with a *χ* ^2^ test. Multivariate logistic regression analysis was performed to determine independent sonographic predictors for lymph node metastasis from the US characteristics that showed statistical significance. Sensitivity, specificity, positive predictive value (PPV), negative predictive value (NPV), and accuracy for each US characteristic suspicious for malignancy were calculated. The diagnostic accuracy of predictions of malignancy was calculated with receiver operating characteristic (ROC) analysis.

## Results

According to the histopathologic examination after thyroid surgery plus prophylactic central lymph node dissection, out of the 712 patients with PTMC, 256 and 456 presented with and without lymph node metastasis, respectively.

The sonographic characteristics of the lymph nodes are shown in Table 1. Compared to nonmetastatic lymph nodes, metastatic lymph nodes were more likely to present the following US characteristics: round shape (41.4 vs. 24.1%), loss of an echogenic fatty hilum (29.7 vs. 1.3%), cystic change (28.9 vs. 8.3%), calcification (34.4 vs. 5.7%) (each *P* < 0.001), and abnormal vascularity (32.8 vs. 22.7%) (*P* = 0.002). However, there were no significant differences between metastatic and nonmetastatic lymph nodes in terms of the US features of boundary (39.5 vs. 35.7%; *P* = 0.326) and echo (37.1 vs. 32.0%; *P* = 0.168).

**Table 1:** The basic characteristics and ultrasound features for the lymph node in PTMCs

Features	Negative LNM (*n* = 456)	Positive LNM (*n* = 256)	*P*
Boundary			0.326
Well defined	293	155	
Poorly defined	163	101	
Echo			0.168
Uniform	310	161	
Nonuniform	146	95	
Shape			<0.001
L/S ratio more than 2	346	150	
Round shape	110	106	
Echogenic fatty hilum			<0.001
Nonloss	450	180	
Loss	6	76	
Cystic change			<0.001
Absent	418	182	
Present	38	74	
Calcification			<0.001
Absent	430	168	
Present	26	88	
Abnormal vascularity			0.002
Absent	354	172	
Present	102	84	

The results of the multivariate logistic regression analysis of the features suggestive of metastatic lymph nodes are shown in Table 2. Five criteria (round shape, loss of echogenic fatty hilum, cystic change, calcification, and abnormal vascularity) were found to be independent factors indicative of metastatic lymph nodes (*P* < 0.05).

**Table 2:** Multivariate analysis of the features suggestive of metastatic lymph nodes

	*P*	HR	95% CI	
			Lower bound	Upper bound
Round shape	0.005	1.750	1.186	2.584
Loss of echogenic fatty hilum	<0.001	15.652	6.377	38.415
Cystic change	<0.001	3.662	2.255	5.946
Calcification	<0.001	4.287	2.476	7.421
Abnormal vascularity	0.035	1.552	1.031	2.336

**Fig. 1:**
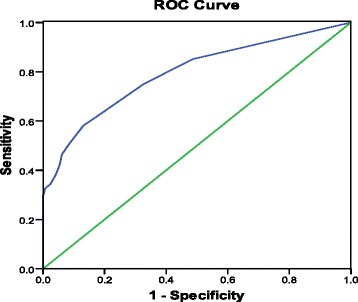
Receiver operating characteristic (ROC) curve for the relationship between ultrasound features and metastatic lymph nodes

The sensitivity, specificity, PPV, NPV, and diagnostic accuracy of the useful sonographic features are shown in Table 3. The respective sensitivity, specificity, and accuracy for the prediction of metastatic lymph nodes were as follows: round shape 41.4, 75.9, and 63.5%; loss of echogenic fatty hilum 29.7, 98.7, and 73.9%; cystic change 28.9, 91.7, and 69.1%; calcification 34.4, 94.3, and 72.8%; and abnormal vascularity 32.8, 77.6, and 61.5%. A receiver operating characteristic curve was calculated to verify the relationship between the number of US features and metastatic lymph nodes (Fig. 1). The area under the curve was 0.793, indicating that the accuracy of the test was good.

**Table 3:** Predictive value of ultrasonography (US) features in thyroid lesions

US feature	Sensitivity (%)	Specificity (%)	PPV (%)	NPV (%)	Accuracy (%)
Round shape	41.4	75.9	49.1	69.8	63.5
Loss of echogenic fatty hilum	29.7	98.7	92.7	71.4	73.9
Cystic change	28.9	91.7	65.5	69.7	69.1
Calcification	34.4	94.3	77.2	71.9	72.8
Abnormal vascularity	32.8	77.6	45.2	67.3	61.5

## Discussion

Sonography has become the imaging method of choice to evaluate cervical lymph node metastasis in patients with PTMC, both for the initial staging and during the subsequent surveillance following thyroidectomy [7, 8]. Moreover, it has been reported that a proportion of patients undergoing reoperation could potentially have been spared from a second surgical procedure shortly after the primary operation due to lateral neck recurrence if lateral neck metastasis had been detected preoperatively [9]. Therefore, early and accurate detection of lymph node metastasis of thyroid cancer and appropriate and prompt treatment thereof are very important in clinical practice.

Normal lymph nodes typically have an oval or elongated shape, with smooth outer contours. The ratio of the long axis (L) to the short axis (S) has been used as an indication of benign morphology. Normal lymph nodes that are reniform or oval have an L/S ratio of >2 [7]. On the other hand, malignant nodes tend to have a rounded morphology and therefore have an L/S ratio of ≤2 [10].

Ahuja et al. suggested that loss of fatty hilum is not a specific ultrasound feature for malignancy [11]. However, in the present study, loss of echogenic fatty hilum was found to be an independent factor to predict cervical lymph node involvement. Furthermore, compared to other US features, loss of fatty hilum had the highest sensitivity and NPV, but lower specificity. This result is consistent with previous reports [12, 13].

Calcifications in the metastatic lymph nodes may be formed by calcification of intravascular tumor thrombi or infarcted tips of malignant papillae [6, 14]. In the present study, the presence of microcalcifications was also found to be a typical sign that suggested cervical lymph node involvement. In accordance with our findings, Rosario et al. reported that calcification had a specificity and PPV of 100%, because this characteristic was not observed in any normal or reactive lymph nodes [8].

Normal cervical lymph nodes may show minimal central hilar vascularity with a branching arborization pattern extending from the echogenic hilum. In many instances, however, normal lymph nodes may appear avascular, without discernible intrinsic vascularity. Metastatic lymph nodes are characterized by any deviation from this normal pattern, with either peripheral flow or a chaotic internal vascular pattern [7]. However, the specificity of resistive indices has not been validated to distinguish benign from malignant nodes [15]. In our study, abnormal vascularity was an independent predicted factor for pathologic lymph nodes.

Some forms of suppurative lymphadenitis can demonstrate intranodal cystic changes; however, fortunately, in patients undergoing surveillance for known thyroid cancer, this condition is not commonly encountered in clinical practice. Cystic changes are shown on ultrasound as small solitary cystic areas, multiple peripheral cystic areas, or almost complete replacement of the node by cystic formation [6]. Kessler et al. reported that cystic changes showed a specificity and PPV of 100% in the diagnosis of metastatic cervical lymph nodes in PTC [16]. Therefore, it can be concluded that cervical lymph nodes in PTC or PTMC are likely to present with cystic changes, and these must hence be considered as pathological changes [7, 17].

In our study, we showed that round shape, loss of echogenic fatty hilum, cystic change, calcification, and abnormal vascularity were independent factors associated with the risk of metastatic lymph nodes. Of note, most of these suspicious US features of loss of fatty hilum, calcification, cystic change, hyperechogenicity, and round shape had high specificity and PPV but low sensitivity and NPV.

Our study has certain limitations. First, this was a retrospective study and included only patients who underwent thyroid surgery or imaging follow-up during a short period. Therefore, selection bias is present. Second, we could not perform a node-by-node analysis of all lymph nodes and, instead, a level-by-level analysis was performed. In addition, only qualitative analyses were performed, and, in future studies, quantitative analyses should also be used. Lastly, the study included a relatively small number of patients. Accordingly, further prospective large-scale studies will be necessary to resolve these issues.

## Conclusions

The results of our study indicate that the US features of round shape, cystic change, calcification, loss of echogenic fatty hilum, and abnormal vascularity are useful sonographic criteria for differentiating cervical lymph nodes with and without metastasis.

## Abbreviations

L: Long axis
NPV: Negative predictive value
PPV: Positive predictive value
PTC: Papillary thyroid cancer
PTMC: Papillary thyroid microcarcinoma
S: Short axis
US: Ultrasonography
